# Unmasking of Brugada syndrome by lamotrigine in a patient with pre-existing epilepsy: A case report with review of the literature

**DOI:** 10.3389/fcvm.2022.1005952

**Published:** 2022-10-28

**Authors:** Hafiz Omer, Mohamed H. Omer, Abdulmohsen R. Alyousef, Ali M. Alzammam, Omar Ahmad, Haitham A. Alanazi

**Affiliations:** ^1^Department of Adult Cardiology, King Abdulaziz Medical City, Riyadh, Saudi Arabia; ^2^College of Medicine, King Saud Bin Abdulaziz University for Health Sciences, Riyadh, Saudi Arabia; ^3^School of Medicine, Cardiff University, Cardiff, United Kingdom; ^4^Department of Internal Medicine, King Abdulaziz Medical City, Riyadh, Saudi Arabia; ^5^College of Medicine, Alfaisal University, Riyadh, Saudi Arabia; ^6^King Abdullah International Medical Research Center, Riyadh, Saudi Arabia

**Keywords:** Brugada syndrome, lamotrigine, epilepsy, syncope, sudden unexpected death in epilepsy (SUDEP), sudden cardiac death (SCD), ion channels

## Abstract

Brugada syndrome is an inherited cardiac channelopathy arising from mutations in voltage-gated cardiac sodium channels. Idiopathic epilepsy portrays a coalescent underlying pathophysiological mechanism pertaining to the premature excitation of neuronal voltage-gated ion channels resulting in the disruption of presynaptic neurons and the unregulated release of excitatory neurotransmitters. The coexistence of epilepsy and Brugada syndrome may be explained by mutations in voltage-gated ion channels, which are coexpressed in cardiac and neural tissue. Moreover, the incidence of sudden unexpected death in epilepsy has been associated with malignant cardiac arrhythmias in the presence of mutations in voltage-gated ion channels. Lamotrigine is an antiepileptic drug that inhibits neuronal voltage-gated sodium channels, thus stabilizing neural impulse propagation and controlling seizure activity in the brain. However, lamotrigine has been shown to inhibit cardiac voltage-gated sodium channels resulting in a potential arrhythmogenic effect and the ability to unmask Brugada syndrome in genetically susceptible individuals. We are reporting a case of a 27-year-old male patient with a background of presumed idiopathic epilepsy who was initiated on lamotrigine therapy resulting in the unmasking of Brugada syndrome and the onset of syncopal episodes. This case provides further evidence for the arrhythmogenic capacity of lamotrigine and highlights the relationship between epilepsy and Brugada syndrome. In this report, we aim to review the current literature regarding the associations between epilepsy and Brugada syndrome and the impact of lamotrigine therapy on such patients.

## Introduction

Brugada syndrome (BrS) is an autosomal dominant channelopathy associated with mutations in voltage-gated ion channels within cardiac myocytes (1). Several genes have been implicated in the development of BrS; however, mutations in the *SCN5A* gene, which codes for the expression of voltage-gated sodium channels, have been correlated with most cases (2). The clinical features of BrS express wide heterogeneity and can range from complete lack of symptoms to malignant ventricular arrhythmias predisposing to sudden cardiac death (SCD) (1). BrS manifests with a characteristic electrocardiogram (EKG) pattern with coved-type ST-segment elevation in the right precordial leads (3). The malignant nature of BrS arises from the presence of arrhythmogenic epicardial substrate in the right ventricular outflow tract, leading to its association with approximately 20% of cases of SCD in individuals with structurally normal hearts (4, 5).

Idiopathic epilepsy portrays a similar pathophysiological mechanism to BrS, with mutations in the neuronal voltage-gated sodium channels implicated in the condition’s development (6, 7). Moreover, some studies have reported the coexistence of idiopathic epilepsy amongst patients with BrS (7). Interestingly, with the conduction of genome-wide association studies amongst patients with idiopathic epilepsy and BrS, some gene mutations coexpressed in both conditions have been identified (8, 9). A recent emerging hypothesis explaining the coexistence of both conditions suggests that the coexpressed genes code for voltage-gated ion channels expressed within cardiac myocytes and neuronal cells (2, 7, 10).

Lamotrigine is an antiepileptic agent that inhibits neuronal voltage-gated sodium channels, stabilizing presynaptic membranes and hindering excitatory neurotransmitter release (11). However, *in vitro* studies have demonstrated that lamotrigine may exhibit class IB antiarrhythmic activity by inhibiting cardiac sodium channels (12, 13). There have been six reports in the literature of lamotrigine contributing to the unmasking of Brugada syndrome, which further supports the theory that lamotrigine may exert a sodium channel-blocking effect within cardiac myocytes (14–19).

In this paper, we aim to report a case of a patient with pre-existing epilepsy who was initiated on lamotrigine therapy resulting in the unmasking of a Brugada pattern on the electrocardiogram. As there have only been six reported cases of lamotrigine unmasking BrS, we aim to contribute to the pre-existing literature on the subject. Moreover, the case also highlights the rare coexistence of BrS with idiopathic epilepsy. Furthermore, we carried out an exhaustive literature review using the Medline database of all reports of lamotrigine-induced BrS as well as reports of the coexistence of epilepsy and BrS.

## Case report

**FIGURE 1:**
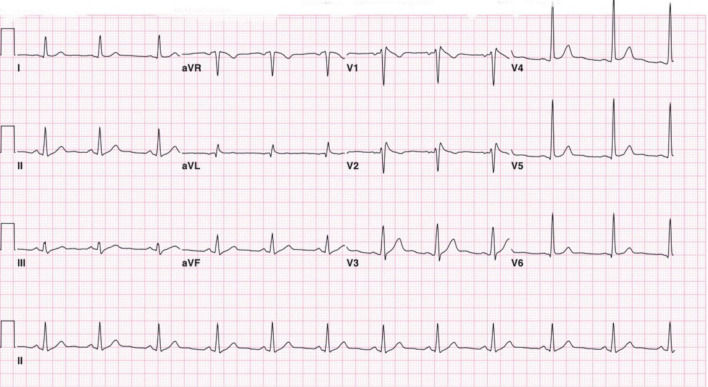
Twelve-lead electrocardiogram demonstrating 2 mm ST segment elevation in leads V1 and V2 with a prominent J wave followed by negative T waves. Suggestive of a type 1 Brugada pattern.

A 27-year-old Saudi-Arabian male with a 4-year history of idiopathic epilepsy was referred to the cardiology department at our hospital for an investigation of his recurrent syncopal episodes and an abnormal electrocardiogram. The patient’s most recent admission was due to a generalized tonic-clonic seizure with lateral tongue biting lasting for 10 min. Seizure activity was rapidly terminated after four milligrams of intravenous lorazepam was administered. The patient recovered immediately with no evidence of confusion or weakness. However, an hour after seizure onset, the patient developed shortness of breath, which lasted for 10 min. A baseline electrocardiogram in the emergency department demonstrated a greater than 2 mm ST segment elevation in leads V1 and V2 with a prominent J wave followed by negative T waves (Figure 1). This was consistent with a type 1 Brugada pattern, facilitating the patient’s referral to our department.

The patient described that he has been experiencing intermittent incidents of transient loss of consciousness preceded by light-headedness and palpitations. Moreover, he reported that this was unusual as he previously experienced one or two episodes annually of generalized tonic-clonic seizures with lateral tongue-biting and urinary incontinence. However, his recent syncopal episodes presented differently as described above and have occurred more frequently. A thorough evaluation of the patient’s family history was undertaken, which did not reveal any history of cardiac disorders, epilepsy, or sudden death. Moreover, the patient described that the incidence of his syncopal episodes began after the initiation of lamotrigine therapy. The patient had previously been prescribed 1,000 milligrams of levetiracetam twice daily; however, 100 milligrams of lamotrigine twice daily was introduced to establish adequate control of seizure activity. Previous electrocardiograms before the initiation of lamotrigine therapy demonstrated normal sinus rhythm with no evidence of a type 1 Brugada pattern.

**FIGURE 2:**
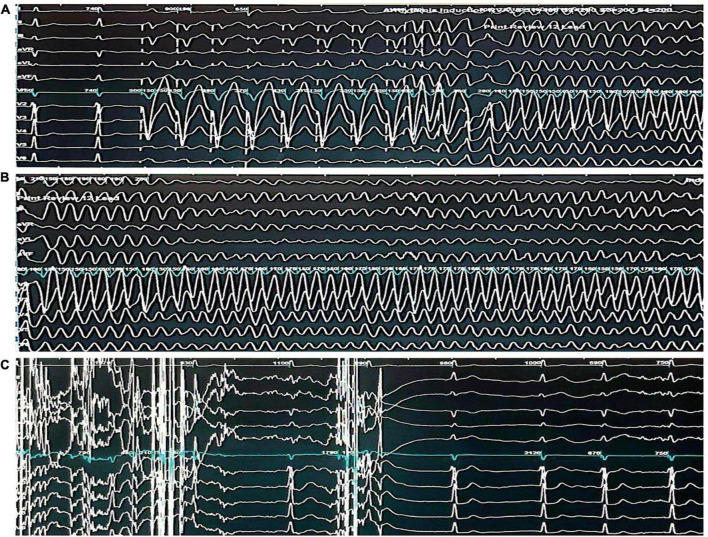
Electrophysiological study demonstrating the induction and termination of ventricular fibrillation. **(A)** Twelve -lead electrocardiogram demonstrating the induction of ventricular fibrillation through triple extrastimuli at the right ventricular outflow tract. **(B)** Twelve-lead electrocardiogram demonstrating sustained ventricular fibrillation. **(C)** Twelve-lead electrocardiogram demonstrating the termination of ventricular fibrillation through a 360-J transthoracic direct current shock and subsequent return to sinus rhythm.

The patient underwent a transthoracic echocardiogram which revealed no evidence of structural heart disease. Further, electrocardiograms were repeated and were consistent with a type 1 Brugada pattern. Moreover, a CT scan of the head revealed no brain abnormalities. An electroencephalogram (EEG) reported during the awake stage revealed well-organized activity with no spike-and-wave discharges or lateralizing abnormalities. Subsequent phonic stimulation did not produce any abnormalities. An electrophysiological study was performed, which resulted in the induction of polymorphic ventricular tachycardia after programmed electrical stimulation of the right ventricular outflow tract (Figure 2). The ventricular tachycardia was terminated through cardioversion, and the patient remained comfortable throughout the study with no hemodynamic compromise.

Due to the presence of a type 1 Brugada pattern on electrocardiography along with a positive electrophysiological study and the presence of syncope, a recommendation for the placement of an implantable cardiac defibrillator (ICD) was made to the patient in accordance with current guidelines (1, 3). A thorough discussion regarding the risk of sudden cardiac death and the utility of ICD implantation was initiated; however, the patient refused further treatment. Moreover, the patient refused to undergo genetic testing to identify deleterious genetic variants in genes coding for voltage-gated sodium channels. A recommendation was made to the neurology team to discontinue lamotrigine therapy due to its potential arrhythmogenic effects.

**FIGURE 3:**
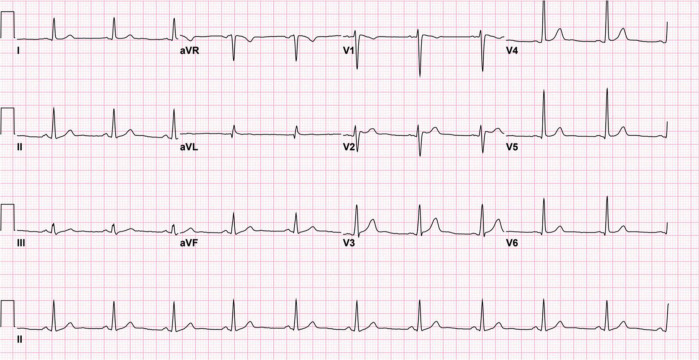
Twelve-lead electrocardiogram after the discontinuation of lamotrigine. The electrocardiogram demonstrates normal sinus rhythm with near resolution of the J-point elevation and type 1 Brugada pattern.

The patient is currently receiving 1,000 milligrams of levetiracetam twice daily and has discontinued lamotrigine therapy after his initial admission. A follow-up assessment 3 months after the patient’s initial admission revealed no further incidences of seizures or syncopal episodes. In addition, further electrocardiograms obtained after the discontinuation of lamotrigine therapy display normal sinus rhythm with near complete resolution of the J-point elevation and the type 1 Brugada pattern (Figure 3).

## Discussion

We are reporting a case of lamotrigine-induced unmasking of BrS in a patient with idiopathic epilepsy. This case highlights two exceedingly rare associations, with one being lamotrigine’s unmasking of BrS, while the other association encompasses the coexistence of epilepsy and BrS amongst specific individuals.

The pathophysiological mechanisms underlying BrS and idiopathic epilepsy share a coalescent theme pertaining to the dysregulation of cardiac and neuronal voltage-gated ion channels resulting in the premature excitability of cells, which manifests as cardiac arrhythmias and seizure-like activity, respectively (1, 6, 7). To identify reports highlighting the coexistence of epilepsy and BrS, we performed a Medline search using the keywords’ Brugada syndrome’, “Epilepsy,” and “Seizure.” Our initial search identified seventy-nine studies, and after abstract screening to identify relevant papers, we identified seventeen publications reporting cases of BrS in association with epilepsy (14–16, 19–32). The essential characteristic features of each paper, including electrocardiogram findings (EKG), electroencephalogram (EEG) findings, features of epilepsy, and the type of antiepileptic therapy, are highlighted in Table 1.

**TABLE 1:** Characteristic features of seventeen publications reporting the association between epilepsy and Brugada syndrome.

First author and year of publication	Patient age and gender	Brugada pattern on EKG	SCN5A mutation status	Epilepsy and EEG features	Antiepileptic medication	Additional information
Abdelghani et al. (20)	30-year-old male	Type 1 Brugada pattern	Not available	- Prior history of transient loss of consciousness with convulsions and epileptiform activity	- Sodium valproate and phenytoin	- Patient presented with cardiac arrest with ventricular fibrillation - Patient underwent epicardial radiofrequency catheter ablation and ICD implantation and was followed-up for 14 months with no further episodes - Antiepileptic medication was discontinued after ablation and ICD implantation
Parisi et al. (21)	- Patient 1: deceased female - patient 2: 40-year-old male - Patient 3: 42-year-old male - patient 4: 5-year-old male patient	All 4 patients had evidence of a type 1 Brugada pattern on EKG	Positive across all four patients	- Patient 1: not available - patient 2: tonic clonic seizures. focal posterior left abnormalities on EEG. - patient 3: tonic clonic seizures. focal posterior left abnormalities on EEG. - patient 4: history of recurrent falls and generalized slow spike-waves on EEG.	- Patient 1: not available - patient 2: phenobarbital - patient 3: phenobarbital - patient 4: levetiracetam, clobazam	- All four patients were members of the same family - All patients had undergone ICD implantation
Sotero et al. (14)	64-year-old male	Type 1 Brugada pattern	Negative	- Focal epilepsy. - EEG showed bilateral focal slow temporal activity and right temporal epileptic activity	- 100 mg Lamotrigine per day - 500 mg of sodium valproate twice daily	- The patient experienced an increase in syncopal episodes after initiation of lamotrigine - EKG prior to initiation of lamotrigine was normal. - Repeat EKG after discontinuation of lamotrigine was normal and flecainide provocative drug test was normal
Fauchier et al. (22)	24-year-old male	Type 1 Brugada pattern	Not available	- History of idiopathic epilepsy with generalized tonic clonic seizures. - One-off EEG was reported as normal.	- Diazepam and phenobarbital	- Patient had witnessed generalized tonic-clonic seizures with evidence of lateral tongue biting and urinary incontinence.
Gülşen and Eker (23)	52-year-old female	Type 1 Brugada pattern	Not available	- Generalized tonic clonic seizures. - EEG revealed bilateral frontal intermittent rhythmic delta wave activity just before the convulsive episode.	- Levetiracetam 1,000 mg per day	- Patient had a paternal history of sudden cardiac death.
Camacho Velásquez et al. (24)	Patient 1: 37-year-old man Patient 2: 49-year-old man	Patient 1: Type 1 Brugada syndrome after Ajmaline provocative drug testing patient 2: type 3 Brugada pattern	Not available in both patients	- Patient 1: tonic clonic seizures with confusion after episodes. No EEG available. - patient 2: tonic clonic seizures. EEG revealed right fronto-temporal spikes	- Patient 1: Valproic acid 1,500 mg daily. - Patient 2: Levetiracetam 2,000 mg per day	- Patient 1 had a positive ajmaline provocative drug test whereas patient 2 had a positive flecainide provocative drug test revealing a Brugada pattern on EKG after infusion
Banfi et al. (15)	30-year-old male	Type 1 Brugada pattern	Negative	Generalized tonic clonic seizures. EEG revealed diffuse slow waves and spikes	- Lamotrigine 3 mg/Kg/day	- The patient had a history of autism spectrum disorder and intellectual disability - The patient was on 15 mg/kg/day of sodium valproate daily prior to being switched over to lamotrigine - EKG findings returned to normal after initiation of lamotrigine - Rare variants identified in SCN9A and AKAP9 genes
Wee and Latorre (25)	25-year-old male	Type 1 Brugada pattern	Not available	EEG revealed diffuse slowing with no epileptiform activity	- Not specified	- Patient was given an anti-epileptic prescription which was never filled. - Patient had previous episodes of loss of consciousness with one episode lasting for approximately 40 min.
Anabtawi et al. (26)	56-year-old female	Type 1 Brugada pattern	Not available	- Not available	- Not specified	
Strimel et al. (19)	22-year-old female	Type 1 Brugada pattern	Not available	- History of temporal lobe epilepsy EEG not available	- 550 mg of lamotrigine daily	- The patient’s lamotrigine blood level was approximately five times the upper limit of the normal range. - Procainamide provocative drug test after normalization of lamotrigine levels demonstrated no appearance of a Brugada pattern on EKG.
Sandorfi et al. (27)	41-year-old male	Type 1 Brugada pattern after procainamide provocative testing	Not available	- History of generalized seizures. - Several EEGs reported as normal, however, continuous EEG monitoring revealed rhythmic seizure activity in the left hemisphere	- Levetiracetam and oxcarbazepine	- The patient underwent ICD implantation
Huang et al. (28)	41-year-old male	Type 1 Brugada pattern	Not available	- Presented to the emergency department in status epilepticus after over half an hour of generalized tonic clonic seizures	- No antiepileptic therapy	- The patient presented with status epilepticus and ventricular fibrillation and direct current cardioversion was delivered which revealed the underlying type 1 Brugada pattern.
Van Gorp et al. (29)	8-year-old male	Type 1 Brugada pattern	Not available	- History of tics, urinary incontinence, and learning disability. Night-time EEG monitoring revealed rapid, rhythmic, and low voltage EEG activity in the left temporal region	- Valproic acid 300 mg twice daily	- This study reported 3-day monitoring of the initiation of antiepileptic medication in a pediatric patient with pre-existing Brugada syndrome.
Ali (30)	59-year-old male	Type 1 Brugada pattern	Not available	- Generalized tonic clonic seizures lasting 30 s with rapid recovery and urinary incontinence	- Not available	- Patient initially presented with ventricular tachycardia - Procainamide provocative drug testing revealed Type 1 Brugada pattern.
Leong et al. (16)	60-year-old female	- Type 1 Brugada pattern after ajmaline provocative drug test	Positive	- Temporal lobe epilepsy - EEG revealed Epileptiform activity in both temporal lobes	- Lamotrigine 125 mg twice daily and 1.5 g of levetiracetam twice daily	
Gigli et al. (31)	40-year-old female	Type 1 Brugada pattern	Negative	- History of juvenile myoclonic epilepsy and recent generalized tonic clonic seizures - EEG revealed generalized spike-wave and spike-slow wave complexes.	- Valproic acid 750 mg per day	- Novel mutation identified in plakophilin 2 (PKP2)
Negro et al. (32)	36-year-old male	Type 1 Brugada pattern after ajmaline administration	Not available	- History of recurrent syncopal episodes with loss of consciousness and convulsions. - EEG not available	- Phenytoin 100 mg per day	- The patient had a family history of epilepsy and sudden unexplained death - Phenytoin levels were recorded to be above therapeutic range - Patient experienced several episodes of ventricular fibrillation and cardiac arrest resulting in the patient’s death - The authors suggest that the epilepsy was misdiagnosed, and the patient had Brugada syndrome which was exacerbated by phenytoin.

Mutations in voltage-gated sodium ion channels underlie the repolarization and depolarization abnormalities observed in BrS (1, 2). Over twenty individual genes have been associated with the development of BrS (2). Notably, loss of function mutations in the *SCN5A* gene, which codes for the alpha-subunit of the Na_*V*_1.5 cardiac voltage-gated sodium channels, have been implicated in over 80% of identifiable mutations in BrS (2–33). Parisi et al. provide a report of a family with a mutation in *SCN5A* with co-expression of BrS and epilepsy along with characteristic EKG and EEG findings which can be found in Table 1 (21). The authors of this paper suggest that individuals who possess the *SCN5A* mutation may have an age-dependent phenotypic expression of this mutation, with cerebral features presenting earlier in life and cardiac manifestations occurring in the later decades. This hypothesis is further supported by patients with gastrointestinal disease harboring the *SCN5A* mutation expressing variable symptoms within each age group (34). Furthermore, Leong et al. highlight a case of a patient harboring an *SCN5A* mutation with temporal lobe epilepsy and BrS (16). Animal studies in rats demonstrate the expression of *SCN5A* genes within the limbic system, suggesting that it can alter neuronal action potential propagation (35). More recently, the expression of *SCN5A* genes within the human brain has been described, with studies suggesting an increased expression within astrocytes and astrocytoma tumors (36–38). Hence, co-expression of *SCN5A* genes within the brain and cardiac tissue may underpin the concurrence of BrS and epilepsy amongst genetically susceptible individuals harboring the *SCN5A* mutation.

Recently, mutations in the *SCN10A* gene, which codes for the voltage-gated sodium channels subunit Na_*V*_1.8, were implicated in a large percentage of BrS cases and were associated with an increased phenotypic expression of symptoms (39). Interestingly, despite the Na_*V*_1.8 being mainly expressed in the peripheral nervous system, in certain pathologies the Na_*V*_1.8 has been found within the central nervous system (40). Moreover, a recent genetic analysis has demonstrated that variants in the *SCN10A* gene were linked to certain epilepsy-related phenotypes (41). Banfi et al. highlight a case of epilepsy associated with novel mutations identified in the voltage-gated sodium channel SCN9A gene, and the *AKAP9* gene, which have previously been implicated in cases of BrS (14, 42). The *SCN9A* gene is also expressed in the brain, with mutations in this gene being associated with the development of certain subtypes of epilepsy (43, 44). It is worth noting that BrS is not the only cardiac channelopathy that has been associated with epilepsy. Namely, the *KCNQ1* gene, which codes for voltage-gated potassium channels, has been correlated with epilepsy and long QT syndrome (LQTS) in a subgroup of patients (45).

Sudden unexplained death in epilepsy (SUDEP) is a phenomenon that carries tremendous morbidity among patients diagnosed with epilepsy. Approximately 18% of patients with epilepsy die due to SUDEP (8). One of the leading theories behind the pathophysiological mechanisms of SUDEP involves the onset of fatal cardiac arrhythmias (42). This theory is supported by retrospective cohort studies which suggest that patients with epileptic seizures have an increased incidence of abnormal electrocardiogram signs (46–48). It is also worth noting that SUDEP and sudden cardiac death have been shown to share similar risk factors, including sex and age (49). Furthermore, some gene mutations in genes that code for cardiac voltage-gated ion channels have been identified in patients with SUDEP. In particular, a few studies have reported mutations of the *SCN5A* and *SCN10A* genes amongst patients with SUDEP (50, 51).

Interestingly, mutations in the *SCN8A* gene, which codes for the alpha subunit type 8 of the voltage-gated sodium channel (Na_*V*_1.6), have been implicated in cases of epilepsy and SUDEP (52). The Na_*V*_1.6 voltage-gated sodium channel is predominantly expressed in the brain; hence mutations in the *SCN8A* gene are associated with a subtype of epilepsy termed early infantile-epileptic encephalopathy-13 (43). Individuals with *SCN8A* mutations possess up to a 10% higher risk of SUDEP (43). A possible explanation for this increased risk relates to the expression of Na_*V*_1.6 in cardiac tissue, with mutations in this gene having been associated with cardiac arrhythmias in animal models (43, 53). Moreover, it is worth noting that the mere presence of the Na_*V*_1.6 channel, under pathological conditions, may contribute to cardiac arrythmias. For instance, an *in vivo* study utilizing mouse models examined the effects of sodium-channel blockade on the induction of catecholaminergic polymorphic ventricular tachycardia (54). The authors of this study noted that blockade of the Na_*V*_1.6 channel reduced the incidence of cardiac arrythmias (54). In addition, the authors hypothesize that Na_*V*_1.6 blockade may interfere with sodium and calcium signaling within cardiac myocytes, thus, specific blockade of Na_*V*_1.6 may provide a novel approach for the treatment of cardiac arrythmias (54). Munger et al. also examined the impact of Na_*V*_1.6 on calcium and sodium channel dysregulation on the induction of atrial fibrillation (55). The authors of this paper suggest that blockade of sodium-channel isoforms such as the Na_*V*_1.6 provides a novel approach for the treatment of atrial fibrillation through modulation of calcium release within myocytes (55). Moreover, the *SCN1A* gene which codes for the Na_*V*_1.1 voltage-gated sodium channel, has also been associated with epilepsy, SUDEP, and cardiac conduction abnormalities across *in vivo* and *in vitro* studies. Mutations in the *SCN1A* lead to the development of Dravet syndrome (a form of early onset severe myoclonic epilepsy) in over 80% of cases (56). The incidence of SUDEP amongst patients with Dravet syndrome is the highest SUDEP rate reported amongst epilepsy subtypes, hence, examining the mechanisms which may underpin SUDEP in Dravet syndrome is essential to further the understanding of the role of sodium channel isoforms in cardiac arrhythmogenesis and epilepsy (57). Auerbach et al. explored the mechanisms behind the altered cardiac electrophysiology and SUDEP in a mouse model with Dravet syndrome (58). The authors of this study noted that the mice with Dravet syndrome exhibited abnormalities in transient and persistent sodium current density within cardiac myocytes (58). The authors also noted the presence of increased cardiac excitability and arrhythmogenic electrocardiogram changes within the Dravet mice (58). Kalume et al. also examined the mechanisms behind SUDEP in a Dravet mouse model (59). The findings of this study suggest that SUDEP in association with *SCN1A* mutations is triggered by parasympathetic overdrive resulting in fatal bradycardia and cardiac dysregulation (59). Experimental models examining the effects of *SCN1A* mutations in epilepsy have described the overexpression of the Na_*V*_1.1 channels within cardiac myocytes and subsequent cardiac hyper-excitability resulting in a predisposition to cardiac arrhythmogenesis (60).

The coexistence of epilepsy and BrS poses a tremendous diagnostic challenge due to the overlap in presentations amongst both diseases. Differentiating true epileptic seizures from arrhythmia-induced syncope amongst patients with BrS is challenging. Seizure-like activity amongst patients with BrS may be a byproduct of cardiac arrhythmias resulting in cerebral hypoperfusion and subsequent syncope (61, 62). There have been four reports of BrS masquerading as a seizure disorder resulting in the misdiagnosis of patients (20, 25, 26, 32). One of the reports by Wee and Latorre was that of a 25-year-old male who was presumed to have epilepsy and presented to the emergency department with an anoxic brain injury resulting in his subsequent death (25). A type 1 BrS pattern was missed on the patient’s previous electrocardiograms; thereby, this report illustrates the significant magnitude associated with a misdiagnosis of epilepsy amongst BrS patients. Moreover, clinical features characteristically associated with epilepsy, such as tongue-biting and generalized tonic-clonic convulsions, may be observed in patients with arrhythmia-induced syncopal episodes, making establishing a final diagnosis a tremendous hurdle (63). The BrS EKG pattern may also not be visible on the initial EKG and may require further assessment through sodium-channel blocker provocative testing. In addition, a multicenter prospective observational study amongst patients with drug-resistant epilepsy found that syncope and epilepsy were coexistent amongst 20% of subjects (64). The discovery of mutations in voltage-gated cardiac ion channels amongst patients with SUDEP may be explained by the existence of potentially undiagnosed cardiac channelopathies and arrhythmias. Misdiagnosis of cardiac channelopathies as epilepsy, is also common with LQTS. A study by MacCormick et al. found that patients diagnosed with epilepsy experienced a delay of approximately 12 years before a correct diagnosis of LQTS was made (65).

Lamotrigine is an antiepileptic drug that inhibits neuronal voltage-gated sodium channels, stabilizing presynaptic neurons and suppressing unregulated glutamate release (11). Recently in 2021, the United States Food and Drug Administration (FDA) issued a warning recommending against using lamotrigine in individuals with structural heart disease and conduction disorders due to its potential arrhythmogenic effect (12). The FDA’s warning was based on *in vitro* studies suggesting that lamotrigine might inhibit cardiac voltage-gated sodium channels with similar pharmacodynamics to class IB antiarrhythmic agents (12, 13). It is well-established that sodium-channel blockers may induce malignant ventricular arrhythmias in patients with BrS (66). Moreover, sodium-channel blocking antiarrhythmic drugs such as flecainide and ajmaline are used to artificially induce ventricular arrhythmias in patients with BrS as part of the diagnostic process (64). An animal study by Goto et al. investigated the effects of lamotrigine therapy on EKG parameters (67). A notable finding from this study included the elevation of the J wave in over half of the animals (67). The J wave is a distinctive feature of BrS, thereby suggesting that lamotrigine may possess the ability to unmask a BrS pattern in genetically susceptible individuals. Lamotrigine exhibits blockade of the brain’s most abundantly expressed voltage-gated sodium channels, including the Na_*V*_1.1, Na_*V*_1.2, and Na_*V*_1.6 (68). However, lamotrigine has also been shown to impact the cardiac voltage-gated sodium channel isoform Na_*V*_1.5 in several *in vitro* studies. In a paper by Ingleby-Talecki et al., lamotrigine was shown to block Na_*V*_1.5 current even at therapeutic dosages (69). Lamotrigine demonstrated a half maximal inhibitory concentration (IC_50_) of 280.2 and 28.8 μM at holding voltages of –120 and –95, respectively (69). Moreover, other *in vitro* studies have reported similar potencies regarding lamotrigine’s Na_*V*_1.5 blockade (13, 70). *In vitro* studies have also shown that lamotrigine demonstrates voltage-dependent blockade of the Na_*V*_1.5 channels with rapid kinetics, closely resembling the class IB anti-arrhythmic agent mexiletine (69). The findings of the aforementioned *in vitro* studies suggest that lamotrigine may be capable of blocking Na_*V*_1.5 channels at plasma concentrations observed within its therapeutic dosage range. Moreover, a human study by Dixon et al. analyzed the effects of lamotrigine on the PR interval in healthy subjects (71). The study demonstrates that lamotrigine may exhibit a dose-dependent prolongation of the PR interval (71). The PR interval may be prolonged due to Na_*V*_1.5 blockade, however, other factors such as heart rate and autonomic stimulation may contribute to PR prolongation (72). The findings of the *in vitro* studies coupled with lamotrigine’s effects on electrocardiogram markers such as the J wave and PR interval in *in vivo* studies suggest that at therapeutic concentrations, lamotrigine may possess the ability to inhibit Na_*V*_1.5 channels and thus contribute to the Brugada phenotype in genetically susceptible individuals.

To explore the association between lamotrigine therapy and the unmasking of BrS, we performed a Medline search using the terms “Lamotrigine” and “Brugada Syndrome.” A total of 11 publications were identified, and abstract screening resulted in the selection of six reports (14–19). The features of each report, including patient characteristics, EKG findings, and lamotrigine dosage, are summarized in Table 2.

**TABLE 2:** Characteristic features of six publications reporting the association between lamotrigine therapy and Brugada syndrome.

Author and year of publication	Patient age and gender	Lamotrigine dose	Brugada pattern on EKG	SCN5A mutation status	Additional information
Sotero et al. (14)	64-year-old male	100 mg per day	Type 1 Brugada pattern	Negative	- Patient had a prior diagnosis of focal epilepsy - The patient was on 500 mg of sodium valproate twice daily and experienced an increase in syncopal episodes after initiation of lamotrigine - EKG prior to initiation of lamotrigine was normal. - Repeat EKG after discontinuation of lamotrigine was normal and flecainide provocative drug test was normal
Banfi et al. (15)	30-year-old male	3 mg/Kg/day	Type 1 Brugada pattern	Negative	- The patient suffered from generalized tonic clonic seizures and had a history of autism spectrum disorder and intellectual disability - The patient was on 15 mg/kg/day of sodium valproate daily prior to being switched over to lamotrigine - EKG findings returned to normal after initiation of lamotrigine - Lamotrigine level was reported to be within the normal range - Rare variants identified in SCN9A and AKAP9 genes
Leong et al. (16)	60-year-old female	125 mg twice daily	No evidence of Type 1 Brugada pattern on initial baseline EKG	Positive	- Patient had a diagnosis of temporal lobe epilepsy - Patient had a family history of sudden cardiac death - The patient was also taking 1.5 g of levetiracetam twice daily - Ajmaline provocative drug test revealed appearance of Type 1 Brugada pattern and QRS widening with bigeminy after 3 min of infusion - Repeat ajmaline provocative drug test after discontinuation of lamotrigine revealed Type 1 Brugada EKG pattern with no QRS widening or bigeminy
Tessitore et al. (17)	62-year-old male	Not available	Type 1 Brugada pattern	Not available	- Patient has a medical history of bipolar disorder and paroxysmal atrial fibrillation - Patient overdosed on 1 gram of Flecainide in addition to an unknown amount of lamotrigine and quetiapine.
Rodrigues et al. (18)	52-year-old woman	100 mg per day	Type 1 Brugada pattern	Not available	- Three EKGS were performed after a flecainide provocative drug test and only one of three revealed a Type 1 Brugada pattern.
Strimel et al. (19)	22-year-old female	550 mg per day	Type 1 Brugada pattern	Not available	- The patient has a history of temporal lobe epilepsy. - The patient’s lamotrigine level was approximately five times the upper limit of the normal range. - Repeat procainamide provocative drug test after normalization of lamotrigine levels demonstrated no appearance of a Brugada pattern on EKG.

Four of the six patients have been treated with lamotrigine for pre-existing epilepsy (14–16, 19). In addition, all patients demonstrated a type 1 BrS pattern on EKG. Interestingly, five patients had a previously normal EKG prior to the initiation of lamotrigine therapy, suggesting that lamotrigine may contribute to unmasking the BrS pattern in susceptible individuals (14–19). Furthermore, the blood levels of lamotrigine were reported to be elevated in a paper by Strimel et al., whereas Banfi et al. reported a lamotrigine level within the normal range (15, 19). The lamotrigine blood level was unavailable within other reports. However, the discrepancy in the lamotrigine blood levels between the two papers highlights that lamotrigine may exhibit an inhibitory effect on cardiac sodium channels at both therapeutic and supra-therapeutic levels. It has previously been hypothesized that lamotrigine may unmask BrS at higher drug concentrations due to loss of specificity for neuronal voltage-gated sodium channels resulting in downstream effects on cardiac voltage-gated channels (19). However, the unmasking of BrS at normal physiological blood levels of lamotrigine contradicts this theory. We hypothesize that individual genetics may interfere with lamotrigine metabolism resulting in significant disparities relating to its pharmacokinetic metabolism amongst different individuals. Lamotrigine exhibits first-order pharmacokinetics and has an excellent oral bioavailability resulting in its rapid absorption with maximal plasma concentrations of the drug occurring within 1–3 h (11). Moreover, the metabolism of lamotrigine is facilitated by different UDP−glucuronosyltransferase enzymes in the liver (11). Individuals express a wide range of heterogeneity amongst genes coding for UDP−glucuronosyltransferases, and multiple polymorphisms can be detected amongst the population (73). The variable pharmacokinetic profile of lamotrigine was investigated in a study of 100 epilepsy patients by Milosheska et al. (74). The study found that multiple factors influenced the clearance of lamotrigine, including genetic polymorphisms in UDP−glucuronosyltransferases, body weight, and renal function (74). Therefore, the authors of this paper suggested that the variability in lamotrigine’s pharmacokinetics governs the need for more precise and individualized drug monitoring and dosage adjustments (74).

As mentioned previously, many patients with cardiac channelopathies have a prior diagnosis of epilepsy requiring antiepileptic drug treatment. The role of antiepileptic drugs in the management of true epileptic seizures is well-established. For instance, the therapeutic interventions used in our patient’s case to manage his epilepsy diagnosis included levetiracetam and lamotrigine, which are supported by clinical and pharmacogenomic studies (75). However, using antiepileptic drugs in patients with non-epileptic seizures and concurrent cardiac channelopathies may result in an arrhythmogenic effect. For instance, a paper by Bardai et al. found an increase in the risk of SCD in association with both epilepsy and the use of antiepileptic drugs (76). Moreover, Ishizue et al. found that polytherapy with multiple sodium channel blocking antiepileptic drugs was associated with arrhythmogenic EKG abnormalities (77). The unmasking of a BrS pattern on EKG has also been observed with other antiepileptic drugs, such as carbamazepine and phenytoin (78, 79).

Nonetheless, the evidence for lamotrigine’s arrhythmogenic effects remains unclear as the FDA’s current recommendation is based solely on the findings of *in vitro* studies. The findings of the aforementioned *in vitro* studies regarding lamotrigine’s impact on cardiac activity have yet to translate clinically. For instance, a recent systematic review by Restrepo et al. explored cardiac risk in patients undergoing lamotrigine therapy and found no clear evidence to support an increase in cardiac risk (80). Therefore, as it currently stands, lamotrigine and other sodium-channel blocking antiepileptic drugs are not contraindicated in patients with BrS.

## Conclusion

As described previously, the coexistence of epilepsy and BrS has been associated with mutations in genes coding for voltage-gated ion channels, which are coexpressed in cardiac and neural tissue (7). However, it is worth noting that the relationship between genotypes and clinical phenotypes is often not linear; hence mutations in genes co-expressed in cardiac and neuronal tissue may independently induce epilepsy and cardiac arrhythmias. Sudden unexplained death in epilepsy has been associated with malignant cardiac arrhythmias due to the presence of mutations in genes coding for voltage-gated cardiac ion channels (7). Consequently, the identification of potentially pathogenic genetic variants which may underlie the pathogenesis of epilepsy, cardiac conduction disorders, and SUDEP requires the use of targeted next-generation sequencing technology and the execution of genome-wide association studies to further our understanding of the genetic basis of the aforementioned conditions (81). Moreover, the coexistence of epilepsy and BrS poses a diagnostic challenge due to the similar nature of clinical presentations. Therefore, we recommend a thorough neurological and cardiovascular evaluation of patients presenting with seizures and syncope to identify any underlying disorders.

Using sodium-channel blocking antiepileptic drugs such as lamotrigine in patients with coinciding epilepsy and BrS may promote arrhythmogenic effects in cardiac myocytes (12). Furthermore, individual genetic polymorphisms may influence the pharmacokinetic profile of lamotrigine, resulting in impaired metabolism and subsequent supratherapeutic blood levels (72). Accordingly, we propose the need for more research to understand the arrhythmogenic capacity of lamotrigine and other antiepileptic drugs. In addition, we recommend the need for precise and individualized therapeutic monitoring and dosage adjustments amongst patients receiving lamotrigine therapy.

## Data availability statement

The original contributions presented in this study are included in the article/supplementary material, further inquiries can be directed to the corresponding author.

## Ethics statement

The studies involving human participants were reviewed and approved by the King Abdullah International Medical Research Center (KAIMRC) Institutional Review Board. Written informed consent was obtained from the participant/s for the publication of this case report.

## Author contributions

MO and AMA wrote the initial manuscript and carried out the literature review process. HO and HA contributed to the acquisition of clinical data and the clinical design of the manuscript. OA contributed to the review of the present literature. AA contributed to the collation of clinical data. All authors reviewed the manuscript and authorized its submission for publication.

## Abbreviations

BrS: Brugada syndrome
SCD: sudden cardiac death
SUDEP: sudden unexplained death in epilepsy
LQTS, long QT syndrome EKG: electrocardiogram
EEG: electroencephalogram
*SCN5A*: Sodium Voltage-Gated Channel Alpha Subunit 5
*SCN10A*: Sodium Voltage-Gated Channel Alpha Subunit 10
*SCN9A*: Sodium Voltage-Gated Channel Alpha Subunit 9
*SCN8A*: Sodium Voltage-Gated Channel Alpha Subunit 8
*AKAP9*: A-Kinase Anchoring Protein 9
*KCNQ1*: Potassium Voltage-Gated Channel Subfamily Q Member 1
FDA: Food and Drug Administration
UDP − glucuronosyltransferases: Uridine 5 ′′ -diphospho-glucuronosyltransferase.
